# Supplementary material for: The heat wave knowledge, awareness, practice and behavior scale: Scale development, validation and reliability
Source: PLoS One. 2022 Dec 21;17(12):e0279259. doi: 10.1371/journal.pone.0279259 (PMC9770401; doi:10.1371/journal.pone.0279259)
Supplement: S1 File — (DOCX) [file pone.0279259.s001.docx]

**Supplementary File 1.**

**SICAK DALGALARI BİLGİ, FARKINDALIK, UYGULAMA VE DAVRANIŞ ÖLÇEĞİ**

**HEAT WAVES KNOWLEDGE, AWARENESS, PRACTICE AND BEHAVIOR SCALE**

| **Item** |  | **Doğru (True)** | **Emin Değilim (Not Sure)** | **Yanlış (False)** |
| --- | --- | --- | --- | --- |
| **K1** | Sıcak dalgaları küresel ısınmanın bir sonucudur.  *Heat waves are a consequence of global warming.* |  |  |  |
| **K2** | Sıcak dalgaları hava kirliliğinden etkilenmez.  *Heat waves are not affected by air pollution.* |  |  |  |
| **K3** | Şehirlerdeki sıcaklıklar çevrelerindeki kırsal ve doğal alanlara göre daha yüksektir.  *Temperatures are higher in cities than in the rural and natural areas around them.* |  |  |  |
| **K4** | Sıcak dalgası kalıcı bir sıcaklık artışıdır.  *A heat wave is a permanent rise in temperature.* |  |  |  |
| **K5** | Sıcak dalgası birkaç gün sürer.  Heat waves last for a couple of days. |  |  |  |
| **K6** | Sıcak dalgaları hastalık ve ölümleri artırır.  *Heat waves rise deaths and diseases.* |  |  |  |
| **K7** | Sıcak dalgasının derecesinden ziyade süresi sağlık sorunlarını daha fazla etkiler.  *The duration of a heat wave has a greater impact on health problems than the degree of a heat wave.* |  |  |  |
| **K8** | Sıcak dalgası sırasında nem, havalandırma vb. ortam faktörleri sağlığı etkilemez.  *During a heat wave, ambient conditions such as humidity and ventilation do not effect the health.* |  |  |  |
| **K9** | Sıcaklığa bağlı ölümlerin maliyeti ve yıkımları çoğu zaman görünür değildir.  *The cost of and destruction caused by temperature-related deaths are often not visible. (overlooked, neglected, unnoticed)* |  |  |  |
| **K10** | Sıcak dalgası görüldüğünde bölgedeki tüm insanlar risk altındadır.  *When a heat wave occurs, all the people in the area are at risk.* |  |  |  |
| **K11** | Çocuklar sıcak dalgasından etkilenmez.  *Children are not affected by a heat wave.* |  |  |  |
| **K12** | Yaşlılar sıcak dalgasından daha fazla etkilenir.  *Elderly individuals are more affected by a heat wave.* |  |  |  |
| **K13** | Gebeler sıcak dalgasından daha fazla etkilenir.  *Pregnant women are more affected by a heat wave.* |  |  |  |
| **K14** | Kronik hastalığı olanlar (Kalp hastalığı, hipertansiyon, şeker hastalığı vb.) sıcak dalgasından daha fazla etkilenir.  *People with chronic diseases (heart disease, hypertension, diabetes, etc.) are more affected by a heat wave.* |  |  |  |
| **K15** | Sıcak dalgaları psikiyatrik hastalıkları tetikler.  *Heat waves trigger psychiatric illnesses.* |  |  |  |
| **K16** | Ekonomik durumu kötü olanlar sıcak dalgalarından daha fazla etkilenir.  *People with a poor economic status may be more affected by heat waves.* |  |  |  |
| **K17** | Sıcak dalgaları su kıtlığına neden olmaz.  *Heat waves cannot cause water shortages.* |  |  |  |
| **K18** | Sıcak dalgaları işçilerin yaralanma ve iş kazası riskini arttırabilir.  *Heat waves can lead to an increased risk of injury and occupational accidents among workers.* |  |  |  |
| **K19** | Sıcak dalgaları orman yangınlarının bir nedeni değildir.  *Heat waves are not a cause of wildfires.* |  |  |  |
| **K20** | Sıcak dalgası sırasında yiyecek ve su kirliliği meydana gelebilir.  *Food and water pollution can be encountered during a heat wave.* |  |  |  |

| **Item** |  | **Kesinlikle Katılmıyorum *(Strongly Disagree)* (1)** | **Katılmıyorum *(Disagree)* (2)** | **Emin Değilim *(Not Sure)* (3)** | **Katılıyorum *(Agree)*  (4)** | **Kesinlikle Katılıyorum *(Strongly Agree)* (5)** |
| --- | --- | --- | --- | --- | --- | --- |
| **A1** | Aşırı sıcaklık olaylarının sıklığı küresel olarak artmaktadır.  *The frequency of extreme temperature events has been increasing globally.* |  |  |  |  |  |
| **A2** | Gelecek yıllarda sıcak dalgalarının daha fazla görüleceğinden endişe duyuyorum.  *I am concerned that there will be more heat waves in the coming years.* |  |  |  |  |  |
| **A3** | Yaşadığım yerde ciddi sağlık etkileri olan sıcak dalgaları görülebilir.  *Heat waves with severe effects on health can occur in the area where I reside.* |  |  |  |  |  |
| **A4** | TV-gazete, internet vb. mecralarda sıcak dalgası konusunda gördüğüm haberler dikkatimi çeker.  *I am interested in news about heat waves on TV, newspapers, internet, etc.* |  |  |  |  |  |
| **A5** | Sıcak dalgasına karşı hazırlıklı olabiliriz.  *We can be prepared for a heat wave.* |  |  |  |  |  |
| **A6** | Devlet kurumları, sıcak dalgaları konusunda etkili önlemler alabilir.  *Government agencies can take effective measures against heat waves.* |  |  |  |  |  |
| **A7** | Eğer yeterince önlem alınmazsa yakın gelecekte sıcak dalgalarına bağlı ciddi sağlık sonuçları görülecektir.  *If adequate measures are not taken, serious health consequences will occur due to heat waves in the near future.* |  |  |  |  |  |
| **A8** | Alınacak tedbirler sıcak dalgalarıyla ilişkili yüksek ölüm oranlarını azaltabilir.  *High mortality rates associated with heat waves can be reduced by certain measures.* |  |  |  |  |  |
| **A9** | Yeşil alanların artırılması sıcak dalgalarından korunmayı sağlamaz.  *Increasing green spaces does not protect against heat waves.* |  |  |  |  |  |
| **A10** | Sıcak dalgaları sırasında toplumu bilgilendirici önlemlere ihtiyaç vardır.  *Measures to inform the community are required during heat waves*. |  |  |  |  |  |
| **B1** | Sıcak dalgalarında risk altındaki kişilere sesli ve yazılı bilgilendirme mesajları gönderilmesi önemlidir.  *It is important to send informative voice messages and text messages to people at risk during heat waves.* |  |  |  |  |  |
| **B2** | Sıcak dalgalarından korunmak için gün içinde şapka/şemsiye kullanırım.  *I use hats/umbrellas during the day to protect myself from heat waves.* |  |  |  |  |  |
| **B3** | Sıcak dalgaları sırasında korunmak için pencereleri kapalı tutarım.  *I keep the windows closed to protect myself during heat waves.* |  |  |  |  |  |
| **B4** | Sıcak dalgası sırasında özellikle öğlen saatlerinde (11:00-15:00) dışarı çıkmaktan kaçınırım.  *During a heat wave, I avoid going out, especially at noon (11:00–15:00 hours).* |  |  |  |  |  |
| **B5** | Sıcak dalgası sırasında fiziksel çaba gerektiren işlerden kaçınırım.  *I avoid physically demanding tasks during a heat wave.* |  |  |  |  |  |
| **B6** | Sıcak dalgaları sırasında bol su içerim.  *I drink a lot of water during heat waves.* |  |  |  |  |  |
| **B7** | Sıcak dalgası sırasında alkol ve kafeinli içeceklerden kaçınırım.  *I avoid alcohol and caffeinated beverages during a heat wave.* |  |  |  |  |  |
| **B8** | Sıcak dalgalarını azaltmak veya önlemek için bireysel düzeyde yapılabilecek çok az şey var.  *There is little that can be done on an individual level to reduce or prevent heat waves.* |  |  |  |  |  |
| **B9** | Sıcak dalgaları sırasında hafif yemekler yenmeli, proteinli gıdalardan kaçınılmalıdır.  *One should eat light meals and avoid foods with protein during heat waves.* |  |  |  |  |  |
| **B10** | Sıcak dalgası olduğu dönemde hava durumunu takip ederim.  *I keep track of the weather when there is a heat wave.* |  |  |  |  |  |
| **B11** | Hava tahmin raporlarını düzenli olarak takip ederim.  *I regularly keep an eye on weather reports.* |  |  |  |  |  |
| **B12** | Sıcak dalgaları konusunda uyarı yapılırsa bildiğim önlemleri uygularım.  *If I am warned about heat waves, I will take the precautions I know.* |  |  |  |  |  |
| **B13** | Sıcak dalgası sırasında resmi kurumların uyarılarına uyarım.  *I follow the warnings of government agencies during a heat wave.* |  |  |  |  |  |
| **B14** | Sıcak dalgası sırasında sağlık çalışanlarının önerilerine uyarım.  *I follow the advice of health workers during a heat wave.* |  |  |  |  |  |
